# Assessing Emotional Responses in People With Dementia Using Facial Assessment Software

**DOI:** 10.1093/geroni/igaf122.594

**Published:** 2025-12-31

**Authors:** Ellen McCreedy, Kelley Hurley, Ann Reddy, Corinne Roma

**Affiliations:** Brown University, School of Public Health, Providence, Rhode Island, United States; CareLink, East Providence, Rhode Island, United States; Brown University, Providence, Rhode Island, United States; Brown University, Providence, Rhode Island, United States

## Abstract

Facial assessment software may help people living with dementia better communicate their emotional states, particularly as the disease progresses, and may help researchers evaluate the effect of promising interventions on momentary affective states. For this pilot study, we partnered with CareLink, a Rhode Island-based healthcare network providing high-quality, evidence-based care for older adults, and SOLO, facial assessment software which has been integrated into a user-friendly application that can be accessed from a tablet or smartphone (https://www.imsolo.ai/). We compared SOLO output to results from Dementia Mood Picture Test (DMPT), a validated self-report tool in which people living with dementia self-report current emotion states using pictures of faces. CareLink enrolled 12 community-dwelling older adults with mild or moderate dementia between September 2023 and April 2024. There were 48 valid comparisons of SOLO and DMPT mood states (mean 4 per participant, range 2-6). Pearson correlation coefficients were computed to assess the linear relationship between self-reported happiness, worry, anger, sadness, and “good mood” and SOLO-detected happiness, stress, anger, sadness, and wellbeing. After controlling for repeated observations, there were moderate positive correlations between self-reported good mood and SOLO-detected well-being, self-reported happiness and SOLO-detected happiness, and self-reported worry and SOLO-detected stress. Sadness and anger were rarely self-reported. CareLink administered both tools and will discuss their acceptability and face validity with patients, care partners and clinical staff. Facial assessment software may be helpful in discerning positive responses to behavioral interventions in people living with dementia.

